# Malignant Airway Stenosis Successfully Treated Using a Combination of Interventional Pulmonology, Chemotherapy, and Radiotherapy

**DOI:** 10.1155/2024/6779155

**Published:** 2024-09-12

**Authors:** Jose N. Sancho-Chust, Anastasiya Torba, Eusebi Chiner

**Affiliations:** Respiratory Department Sant Joan d'Alacant University Hospital, Sant Joan d'Alacant, Spain

## Abstract

Interventional pulmonology can be helpful in cases of malignant airway stenosis. We present a 73-year-old man diagnosed with lung cancer who presented with symptomatic airway stenosis caused by a large endobronchial tumor. Oncological treatment was started with chemotherapy, radiotherapy, and a multimodality bronchoscopic approach using balloon bronchoplasty, electrosurgery, and argon plasma coagulation. Response evaluation showed relief of symptoms, disappearance of the endobronchial tumor, and complete resolution of the airway stenosis.

## 1. Introduction

Interventional pulmonology with therapeutic bronchoscopy is used to treat airway stenosis in lung cancer patients. Therapeutic bronchoscopic tools can provide relief of symptoms such as dyspnea or hemoptysis and achieve improvements in bronchial stenosis [1].

## 2. Case Presentation

We present a 73-year-old male, a former smoker with a cumulative smoking rate of 50 pack-years, referred to the respiratory medicine clinic with a chief complaint of dyspnea on exertion (Modified Medical Research Council (mMRC) Dyspnea Scale Grade 2) and dry cough. A chest X-ray showed a pulmonary mass in the upper right lobe.

**Figure 1:**
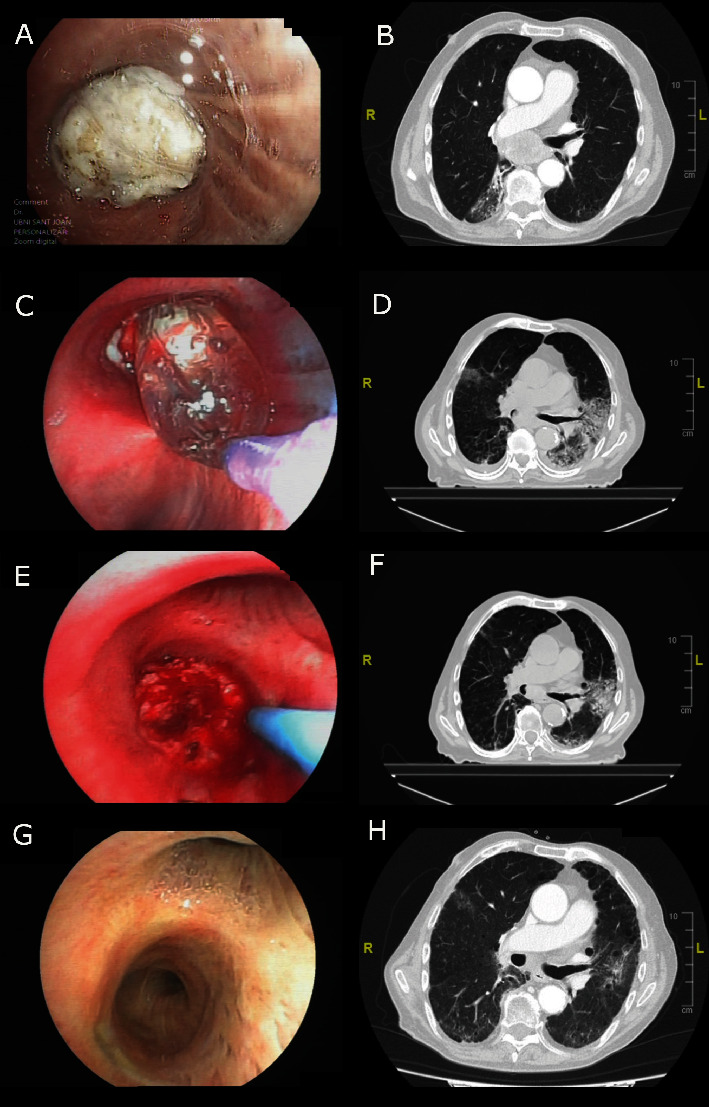
(A) Initial diagnostic bronchoscopy showing a large endobronchial tumor causing a total obstruction of the right main stem bronchus. (B) Initial CT scan with a large subcarinal mass. (C) First therapeutic bronchoscopy with balloon bronchoplasty. (D) CT scan after the start of systemic treatment and bronchoplasty with a slight permeabilization of the bronchial lumen and a decrease in the size of the subcarinal mass. (E) Second therapeutic bronchoscopy when bronchial electrosurgery and argon plasma coagulation (shown in the picture) were applied. (F) Subsequent follow-up CT scan with a recovery of 50% of the lumen of bronchus intermedius and a decrease in the size of the subcarinal mass. (G) Final bronchoscopic evaluation showing total regression of the endobronchial tumor with a preserved bronchial lumen of bronchus intermedius. (H) Final CT scan with a maintained decrease in size of the subcarinal mass.

Initial diagnostic bronchoscopy showed complete right main stem bronchus obstruction by an endobronchial tumor (Figure 1(A)). Initial diagnostic CT scan showed a peripheral mass in the right upper lobe with homolateral mediastinal involvement including a large subcarinal mass causing total obstruction of the bronchus intermedius (Figure 1(B)).

The patient went into respiratory failure and started treatment with supplementary oxygen delivered by nasal prongs at a flow rate of 3 L/min. He was diagnosed with squamous cell carcinoma of the lung (cT2aN2M0, Stage IIIA), and after a multidisciplinary assessment, systemic treatment with a concomitant radiochemotherapy schedule was planned. Initial chemotherapy included carboplatin (area under the curve [AUC] 5 mg/mL min) and paclitaxel (175 mg/m^2^) for 4 weeks.

In the second week of treatment, balloon bronchoplasty by flexible bronchoscopy was applied (Figure 1(C)). Follow-up CT scan showed a decrease in the size of the mediastinal mass and a slight bronchial lumen in the bronchus intermedius (Figure 1(D)).

In the fourth week of systemic treatment, a second therapeutic flexible bronchoscopy was performed. Bronchial electrosurgery with an electrocautery snare achieved partial resection of the endobronchial tumor, and argon plasma coagulation was used for devitalization of the remaining tumor (Figure 1(E)). A CT scan confirmed the recovery of 50% of the lumen of the bronchus intermedius (Figure 1(F)).

Radiotherapy was started at the fifth week of treatment with volumetric modulated arc therapy-image guided radiotherapy (VMAT-IGRT), with a total dose of 66 Gy in 33 fractions for 6 weeks. During the same period, chemotherapy with paclitaxel was applied weekly. The patient completed 10 weeks of systemic treatment without any reported side effects.

Response evaluation at 6 months was performed. Clinically, the patient did not report cough or dyspnea (mMRC Grade 0), with decreased supplementary oxygen requirements and a reduction of the flow rate to 2 L/min. Bronchoscopy showed total regression of the endobronchial tumor with a preserved bronchial lumen of the bronchus intermedius (Figure 1(G)). In the CT scan, the decrease in size of the subcarinal mass was maintained (Figure 1(H)).

## 3. Discussion

Therapies in patients with lung cancer are based on a multimodal treatment, including local therapies such as surgery and radiotherapy and systemic therapies with a wide-ranging option of chemotherapies and novel immunotherapies [2].

Many patients with lung cancer develop airway obstruction. The most frequent clinical features are dyspnea, hemoptysis, secretions, and recurrent pneumonia, decreasing the quality of life of these patients. Unfortunately, only a small part of these patients will be amenable to curative surgery. When surgery is not a choice, several noninterventional palliative therapies can be used to relieve the suffering experienced by the patients and their caregivers. If these measures are not enough, interventional palliative therapies can be offered by pulmonologists with the application of interventional pulmonology [3, 4].

In its therapeutic approach, interventional pulmonology includes many therapies such as bronchial stenting, rigid bronchoscopy coring, argon plasma coagulation, laser, electrosurgery, cryotherapy, photodynamic therapy, or brachytherapy [5, 6].

The choice of intervention is usually influenced by the type of airway involvement. Mechanical debridement or ablative therapy is most appropriate for endobronchial tumors, while bronchoplasty or stenting is best suited for extrinsic compression. Another relevant factor is the presence of viable lower airways and distal lungs, which may influence whether intervention is appropriate or not. Other factors to be considered include patient characteristics, resource availability, and local expertise [7, 8].

Interventional pulmonology has shown benefits in relieving symptoms and improving the quality of life of these patients, also with benefits in terms of cost-effectiveness [9, 10].

Balloon bronchoplasty can be used to treat malignant airway stenosis, achieving immediate bronchial dilatation. It can be applied using both rigid and flexible bronchoscopy. However, its effect is usually short-lived and requires repeated applications or the addition of other interventional procedures [7, 11].

Bronchial electrosurgery is a modality of ablative therapy that uses an electrical probe to conduct an electrical current to directly heat tissue, providing a safe and effective way to cut endobronchial tumors [12, 13].

Argon plasma coagulation is safe and effective in treating large surface areas of mucosa in malignant endobronchial lesions, showing benefits in terms of tumor devitalization and hemostasis [14, 15].

In our case, balloon bronchoplasty was first chosen to achieve a minimum bronchial lumen on which to subsequently apply a combined ablative and devitalizing therapy with bronchial electrosurgery and argon plasma coagulation. These interventional pulmonology procedures, in combination with systemic oncological treatment, allowed the unusual endobronchial tumor disappearance and the complete resolution of the airway stenosis.

In conclusion, the documented bronchoscopic and radiological evolution of the presented case underlines the usefulness of interventional pulmonology as part of a multimodal treatment of lung cancer patients with airway stenosis. Therefore, interventional pulmonology procedures should be considered in these patients when curative surgery is not feasible.

## Data Availability

Data sharing is not applicable to this article as no new data were created or analyzed in this study.

## References

[1] Avasarala S. K., Rickman O. B. (2022). Endobronchial therapies for diagnosis, staging, and treatment of lung cancer. *The Surgical Clinics of North America*.

[2] Petrella F., Rizzo S., Attili I. (2023). Stage III non-small-cell lung cancer: an overview of treatment options. *Current Oncology*.

[3] Ali M. S., Sorathia L. (2018). Palliative care and interventional pulmonology. *Clinics in Chest Medicine*.

[4] Feller-Kopman D. (2020). Interventional pulmonology: there is no going back, only forward. *Respirology*.

[5] Tofts R. P., Lee P. M., Sung A. W. (2013). Interventional pulmonology approaches in the diagnosis and treatment of early stage non small cell lung cancer. *Translational Lung Cancer Research*.

[6] Kalsi H. S., Thakrar R., Gosling A. F., Shaefi S., Navani N. (2020). Interventional pulmonology: a brave new world. *Thoracic Surgery Clinics*.

[7] Wahidi M. M., Herth F. J. F., Chen A., Cheng G., Yarmus L. (2020). State of the art: interventional pulmonology. *Chest*.

[8] Agrawal A. (2021). Interventional pulmonology: diagnostic and therapeutic advances in bronchoscopy. *American Journal of Therapeutics*.

[9] Pastis N. J., Silvestri G. A., Shepherd R. W. (2013). Quality-of-life improvement and cost-effectiveness of interventional pulmonary procedures. *Clinics in Chest Medicine*.

[10] Amjadi K., Voduc N., Cruysberghs Y. (2008). Impact of interventional bronchoscopy on quality of life in malignant airway obstruction. *Respiration*.

[11] Karakoca Y., Karaagac G., Aydemir C. (2009). Therapeutic bronchoscopic intervention with resector balloon. *Journal of Bronchology & Interventional Pulmonology*.

[12] Mahajan A. K., Ibrahim O., Perez R., Oberg C. L., Majid A., Folch E. (2020). Electrosurgical and laser therapy tools for the treatment of malignant central airway obstructions. *Chest*.

[13] Chaddha U., Hogarth D. K., Murgu S. (2019). Bronchoscopic ablative therapies for malignant central airway obstruction and peripheral lung tumors. *Annals of the American Thoracic Society*.

[14] Sancho-Chust J. N., Cases Viedma E., Martinez Tomas R., Chiner V. E. (2019). Argon plasma coagulation for management of hemoptysis in endobronchial metastasis from soft-tissue sarcoma. *Respiratory Medicine Case Reports*.

[15] Gesthalter Y. B., Channick C. L. (2024). Interventional pulmonology: extending the breadth of thoracic care. *Annual Review of Medicine*.

